# Rare variants of *GPSM2* associated with aneuploidy-mediated recurrent spontaneous abortion: mechanistic insights from siRNA knockdown modeling of hypothesized loss-of-function

**DOI:** 10.3389/fcell.2026.1677758

**Published:** 2026-06-17

**Authors:** Kai Liao, Yijian Zhu, Yuwei Zhao, Yi Liu, Hongyan Chen, Daru Lu

**Affiliations:** 1 State Key Laboratory of Genetics and Development of Complex Phenotypes and MOE Engineering Research Center of Gene Technology, School of Life Sciences and Taizhou Institute of Health Science, Fudan University, Shanghai, China; 2 The Central Laboratory of Birth Defects Prevention and Control, Ningbo Key Laboratory for the Prevention and Treatment of Embryogenic Diseases, Ningbo Key Laboratory of Genomic Medicine and Birth Defects Prevention, The Affiliated Women and Children's Hospital of Ningbo University, Ningbo, China; 3 NHC Key Laboratory of Birth Defects and Reproductive Health, Chongqing Population and Family Planning Science and Technology Research Institute, Chongqing, China; 4 Department of Gastroenterology, Huashan Hospital, Fudan University, Shanghai, China

**Keywords:** aneuploidy, G protein signaling modulator 2, nuclear mitotic apparatus, recurrent spontaneous abortion, whole-exome sequencing

## Abstract

**Introduction:**

Recurrent spontaneous abortion (RSA) is associated with embryonic chromosomal abnormalities, yet the maternal genetic factors predisposing to the production of aneuploid gametes remain incompletely understood. Identifying germline variants that disrupt meiotic chromosome segregation may elucidate the genetic etiology of RSA.

**Methods:**

Peripheral blood DNA was collected from 100 RSA patients for whom aneuploidy of embryonic origin had been confirmed in prior miscarriage specimens, and whole-exome sequencing (WES) was performed. Rare missense variants in candidate meiotic genes were identified and prioritized for functional analysis. The effect of GPSM2 loss-of-function was modeled via siRNA-mediated knockdown in germ cell lines, and meiotic progression, ploidy outcomes, and spindle-associated protein localization were assessed in vitro and in vivo.

**Results:**

Two patients harbored rare heterozygous missense point mutations in GPSM2 (G protein signaling modulator 2). GPSM2 knockdown resulted in a marked increase in diploid spermatocytes (NC: 10.6% vs. GPSM2-siRNA: 53.8%) and a corresponding decrease in haploid spermatids (NC: 57.8% vs. GPSM2-siRNA: 5.49%), indicating failure of the first meiotic division, while earlier spermatogenic stages remained unaffected. Mechanistically, GPSM2 was found to interact with the nuclear mitotic apparatus (NUMA) protein; upon GPSM2 depletion, chromosome separation was arrested at metaphase in GC-2 cells, and NUMA failed to localize to the spindle poles.

**Discussion:**

These findings suggest that rare GPSM2 missense mutations predicted to impair gene function may compromise first meiotic chromosome segregation through disruption of the GPSM2–NUMA interaction at the spindle pole, thereby predisposing carriers to produce aneuploid germ cells. Fertilization of such aneuploid gametes would yield chromosomally abnormal embryos, providing a mechanistic basis for RSA in these patients. However, definitive causal attribution awaits direct functional characterization of the specific missense variants identified, as the current study employed siRNA-mediated knockdown as a surrogate loss-of-function model.

## 1 Introduction

Recurrent spontaneous abortion (RSA) refers to the loss of two or more pregnancies. RSA is a gynecological complication with a high incidence that has a serious impact on the physical and mental health of women. Approximately 15% of clinically diagnosed pregnancies end in miscarriage, the majority of which occur during the first trimester. The probability of having two consecutive miscarriages is 1%–5%, and the probability of having more than three consecutive miscarriages is less than 1% (Bender Atik et al., 2018; Practice Committee of the American Society for Reproductive Medicine, 2012). The etiology of RSA includes genetic and nongenetic factors. The genetic factors are mainly chromosomal abnormalities in the products of conception (POC), which account for approximately 29%–50% of RSAs. Routine cytogenetic analysis indicates that 50% of these cases are caused by chromosomal abnormalities, such as aneuploidy (Hassold et al., 1980).

Whole-exome sequencing (WES) targets the protein-coding regions of DNA. The exome accounts for 1% of the human genome and is estimated to contain 85% of the gene mutations associated with disease (Choi et al., 2009). Compared with whole-genome sequencing (WGS), WES is less expensive, generates smaller datasets, and is more manageable while still providing comprehensive coverage of the coding regions of DNA. WES has been widely used in clinical practice to detect genetic factors for structural anomalies, third-trimester pregnancy failure, and developmental disorders (Carss et al., 2014; Wright et al., 2015; Shamseldin et al., 2018). However, a few WES studies have reported that gene mutations may lead to RSA. *KIF14* is a compound heterozygous mutation in an unexplained euploid RSA family (Filges et al., 2014). Meanwhile, a homozygous *ECEL1* missense mutation has caused arthrogryposis multiplex congenita and terminations in close relatives in a family (Dohrn et al., 2015). A novel homozygous *MUSK* mutation was identified in an independent couple with a history of fetal akinesia and RSA (Wilbe et al., 2015). Couples with RSA related to fetal microcephaly carry *STIL* compound heterozygous mutations (Cristofoli et al., 2017), and a homozygous nonsense mutation in the *CEP55* gene was detected in a non-consanguineous couple with two fetuses, both with Meckel syndrome (Bondeson et al., 2017). Couples experiencing fetal abnormalities and recurrent abortion were found to carry *IFT122* heterozygous mutations (Tsurusaki et al., 2014). A separate study revealed that two consanguineous Iranian couples who experienced RSA carried homozygous missense mutations in *NOP14* (Suzuki et al., 2018). Two WES studies of RSA caused by fetal edema were the first to report that men in a conventional couple with *FOXP3* gene mutations caused disease in a pair of multiple pregnancy failures (Rae et al., 2015). *FOXP3* is an X-linked gene known to cause fetal idle syndrome (Rae et al., 2015). The second novel mutation was identified in a consanguineous couple, with the autosomal recessive mutation of *CHRNA1* also causing fetal anemia (Shamseldin et al., 2013).

No studies have reported that recurrent miscarriage caused by aneuploid embryos is attributed to genetic mutations in patients. The above studies did not focus on RSA caused by aneuploid embryos. Therefore, we collected peripheral blood from 100 individuals with abnormal chromosomal contributions to RSA caused by aneuploid embryos from our previously reported program (Lei et al., 2023) and performed WES analysis. Two patients carried rare point mutations in G protein signaling modulator 2 (*GPSM2*), whose pathogenicity was predicted using software. Amino acid synthesis controlled by *GPSM2* was also conserved in the evolutionary sequence of each species. GPSM2 is a conserved protein that participates in the regulation of cell polarity and spindle organization during mitosis and meiosis (Zhu et al., 2011). During the interphase of mitosis, the GPSM2 protein is distributed in the cytoplasm and is spatially separated from the nuclear mitotic apparatus (NUMA) protein; the GPSM2 protein interacts with microtubules and plays a role in the formation and organization of the mitotic spindle during cell division. However, in the middle and late stages of mitosis, this protein colocalizes with the NUMA protein at the spindle poles. Some researchers suggest that the C-terminal of the GPSM2 protein interacts with the N-terminal of the NUMA protein to form a complex, while NUMA simultaneously associates with the dynein/dynactin motor complex. Under the guidance of the GPSM2 protein, the entire complex protein moves towards the negative end of the spindle, pulling the microtubules together to form the spindle poles, guiding the chromosomes to move towards the two poles of the cell, and completing the correct separation of the chromosomes (Zhu et al., 2011). The overexpression or RNA interference of GPSM2 during the first round of meiotic division in mice disrupts the spindle pole structure, leading to incorrect chromosome separation (Du et al., 2001; Guo and Gao, 2009). Experiments in cell and animal models have shown that *GPSM2* knockdown can lead to abnormalities in the first stage of meiosis in oocytes, arrest of the first stage of meiosis in mouse spermatocytes, and metaphase arrest of chromosome separation in GC-2 cells. The pathogenesis may be caused by the inability of the NUMA protein to interact with sufficient GPSM2 protein to localize correctly at the poles of the spindle. This study indicates that, if the *GPSM2* mutations lead to the impairment of the gene function, they may result in an increased rate of aneuploidy.

## 2 Materials and methods

### 2.1 Participants

Our research group previously reported the origin of incorrect chromosomes in aneuploid embryo abortion samples from 100 patients with RSA who were recruited from the Shanghai Ji Ai Genetics and IVF Institute and the Obstetrics and Gynecology Hospital of Fudan University (Lei et al., 2023). The study regarding the cohorts was approved by the institutional review boards of all participating institutes, and signed informed consent was obtained from all subjects who participated in the study.

### 2.2 Whole-exome sequencing

Blood DNA was extracted using a TIANamp Blood DNA Kit (TIANGEN). We used 1 μg of genomic DNA to enrich the human exome using the Agilent SureSelect XT Human All Exon Kit and sequenced the DNA on the Illumina HiSeq X-TEN sequencing platform (Illumina). The raw data were mapped to the human genome assembly GRCh37/hg19 using Burrows–Wheeler Aligner (BWA) software. In this study, PCR duplicates were removed using Picard software, and the quality of mutations was assessed by obtaining valid reads, valid bases, average coverage depth, and coverage ranging from 90 to 1,203. ANNOVAR software was subsequently used for functional annotation, with information from a variety of databases and bioinformatics tools, including OMIM and the 1000 Genomes Project. Deleterious missense mutations were predicted simultaneously via SIFT and PolyPhen-2. Sanger sequencing was conducted for mutation verification using the primers listed in Supplementary Table S1. The WES data have been uploaded to the China National GenBank under project number CNP0008044.

### 2.3 Mouse culture

Sexually mature male and female ICR mice (over 4 weeks old) were purchased from Shanghai Jiesjie Laboratory Animal Co., Ltd. (Shanghai, China). The mice were placed in a specific pathogen-free animal room under controlled conditions (temperature, 25 °C ± 2 °C; humidity, 50%–60%; and a 12/12 h light/dark cycle). All mice were acclimated to the housing environment for at least 1 week before formal experiments were performed. For studies involving animals, the animal study protocol was approved by the Laboratory Animal Ethics Committee, School of Life Sciences, Fudan University (protocol number 2022JS071 and approval date 18 May 2022).

### 2.4 Seminiferous tubule injection and microinjection

ICR mice were anesthetized by intraperitoneal injection (sodium pentobarbital). On the micro-operation table, the testes of the mice were exposed to surgical scissors and tweezers, and *GPSM2* siRNA (sense 5′-3′GGU​CUG​AGC​UAC​AGC​ACA​ATT; antisense 5′-3′UUG​UGC​UGU​AGC​UCA​GAC​CTT) (60 µg) or GPSM2 negative control siRNA (sense 5′-3′ UUC​UCC​GAA​CGU​GUC​ACG​UTT; antisense 5′-3′ACG​UGA​CAC​GUU​CGG​AGA​ATT) (60 µg), mixed with Entranster *in vivo* (Engreen Biosystem, Beijing, China) solution supplemented with bromophenol blue as an indicator, was injected into the seminiferous tubules of the mice. After the operation was completed, the wounds of the mice were sutured. A total of 5–10 pL of *GPSM2* negative control siRNA (1 mM; NC) or *GPSM2* small interfering RNA (siRNA) (25 mM) was microinjected into the cytoplasm of the oocytes with an Eppendorf microinjector (Hamburg, Germany), with the procedure completed within 1 h. After injection, the oocytes were cultured for 24 h in M2 medium (Sigma) supplemented with milrinone (Jinpan). They were then transferred to fresh medium and cultured under mineral oil at 37 °C in an atmosphere of 5% CO_2_. After siRNA injection into the testicular seminiferous tubules of the mice for 14 days, we collected samples in a uniform manner for subsequent experiments.

### 2.5 siRNA transfection and synchronization

The cells were seeded into six-well plates 1 day earlier to maintain a cell density of 60%–80%. Afterward, 125 μL of DMEM without antibiotics or serum was added to the EP tubes, followed by 100 pmol of siRNA. The solution was gently mixed by pipetting, after which 4 μL of Lipo 8000™ (Beyotime, Shanghai, China) transfection reagent was added. The solution was mixed again and incubated at room temperature for 20 min. Drops were added to six-well plates for cellular siRNA interference. Some cells were used for qRT‒PCR (Supplementary Table S2) to determine the efficiency of *GPSM2* interference. For synchronization, after 16 h of siRNA interference, the cells were cultured with 10 mM thymidine (Tsbiochem, Shanghai, China) for 16 h. Then, we released the thymidine to replace the medium, and immunofluorescence observation was performed 10 h later.

### 2.6 Immunofluorescence and HE staining

The sections or cell slides were subsequently washed with 1X PBS. They were fixed with 4% paraformaldehyde (Solarbio, Beijing, China) for 30 min at room temperature and then washed three times with 1X PBS for 3 min per wash. The membranes were permeabilized with Triton X-100 for 30 min at room temperature and then washed three times with PBS for 3 min each. The cells were blocked overnight at 4 °C in 3% BSA (Solarbio, Beijing, China). The primary antibody was diluted, incubated at 37 °C for 1 h, and then washed three times with 1X PBS for 5 min per wash. Similarly, the secondary antibody was diluted, incubated at 37 °C for 1 h, and then washed three times with PBS for 5 min per wash. Nuclear staining was performed with DAPI (Beyotime, Shanghai, China), and the cells were observed using a Nikon A1.

The mice were killed by cervical dislocation, and the testicular tissue was removed and cut into small pieces of 2–3 mm thickness to facilitate penetration of the fixative. The removed testicular tissue was rinsed with 1X PBS solution, then fixed with testicular tissue fixation solution (Shanghai Sibao Biotechnology Co., Ltd., Wuhan, China), and sent to Sibao Biotechnology Company (Wuhan, China) for terminal deoxynucleotidyl transferase dUTP nick-end labeling (TUNEL), peptide nucleic acid (PNA), and hematoxylin–eosin staining (HE).

### 2.7 Flow cytometry experiments

The testes were removed, precooled, and washed once with 1X PBS (Solarbio, Beijing, China). We removed the tunica albuginea of the testis and fully chopped the testicular tissue before placement into an EP tube. Type II collagenase (Life-iLab, Shanghai, China) was added to 1 mL of DMEM/F12 to a final concentration of 1.5 mg/mL, and the chopped testicular tissue was transferred at 37 °C and digested with shaking at 50 r/min for 20 min. Then, the supernatant was removed by centrifugation at 1,500 r/min for 5 min. Type II collagenase and hyaluronidase (MERCK, Kenilworth, NJ, United States) were added to 0.5% trypsin to a final concentration of 1.5 mg/mL. We added 500 µL to resuspend the cell mass, followed by digestion for 20–30 min at 37 °C with shaking at 150 r/min. Digestion was terminated by the addition of 500 µL of DMEM/F12 and centrifugation at 1,500 r/min for 5 min to remove the supernatant. We added 500 µL of PBS [containing RNase A (50 μg/mL)], followed by incubation at 37 °C for 30 min and centrifugation at 1,500 r/min for 5 min to remove the supernatant. The samples were subsequently washed once with PBS and centrifuged at 1,500 r/min for 5 min to remove the supernatant. The following working mixture of Triton X-100 + PI (Biosharp, Shanghai, China) was added: PI to a final concentration of 50 μg/mL and Triton X-100 (Sigma‒Aldrich, Saint Louis, MO, United States) to a final concentration of 0.03%, after which the cells were gently mixed and stained for 10 min at room temperature in the dark. After all treatments were completed, the mixture was filtered through a 200-mesh filter and analyzed using the instrument.

### 2.8 Quantification and statistical analysis

Immunofluorescence signal quantification was performed using ImageJ (version 1.52a) software. Data are shown as the mean ± s.d. For comparative analysis, an unpaired Student’s two-tailed t-test was used, and the results were graphed using GraphPad Prism 9.

### 2.9 Principles for screening candidate genes causing diseases

Genes with identical abnormal mutations detected in both WES analyses, present in families with more than two sporadic cases and a minor allele frequency (MAF) of less than 1%, were prioritized. Based on the literature and databases (SIFT, PolyPhen-2, and PROVEAN) for pathogenicity prediction, genes related to “aneuploidy,” “abnormal meiotic division of sperm and eggs,” “abnormal development from fertilized egg to embryo,” and other phenotypes that may cause RSA were selected. In the WES results, loss-of-function mutations such as premature termination and frameshift mutations (variable splicing changes) were prioritized. Candidate genes that have been studied during meiosis, such as *FLNA* and *KNL1*, were not included in the scope of this research. Therefore, we selected the top-ranked candidate pathogenic gene *GPSM2* for our study (Supplementary Table S3).

## 3 Results

### 3.1 Two patients with RSA carried rare mutations in *GPSM2*

**FIGURE 1:**
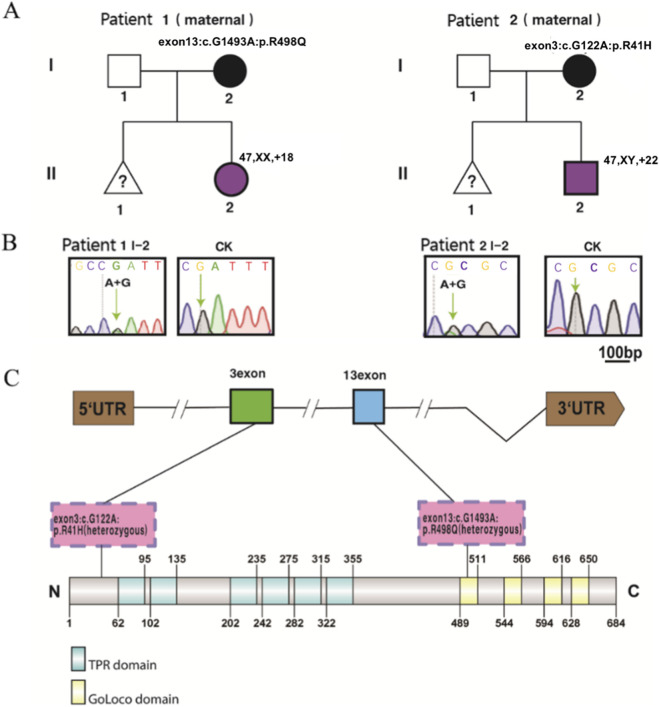
Two mothers with abnormal chromosomes in aneuploid abortions carried rare missense single mutations in *GPSM2*. **(A)** Pedigrees of patients 1 and 2. **(B)** Validation of Sanger sequencing results. A triangle with a question mark indicates that the karyotype of the miscarriage is unknown; black indicates the source of the abnormal chromosome in the abortion product. Purple indicates that the karyotype of the abortion product is known. **(C)** *GPSM2* protein structure and mutation sites. Distribution of missense mutations in the transcripts and primary amino acid sequences of *GPSM2* carried by patients 1 and 2.

The CNV-seq results of the last abortion samples of Patient 1 and Patient 2 were “47,XX+18” and “47,XY+22,” respectively (Supplementary Figure S1). STR results revealed that the abnormal chromosomes all originated from the mother (Figure 1A) (these data have been previously reported) (Lei et al., 2023). According to the STR loci on the abnormal chromosomes, the pregnancy product was compared with those of the couple, and the extra chromosomes 18 and 22 were most likely of maternal origin. They were found to carry rare monoallelic missense point mutations in the G protein signaling modulator 2 (*GPSM2*) gene (Supplementary Tables S3, S4). WES results for Patient 1 are provided in Data Sheet 2 (WES ID: WES-190021958), who harbors the *GPSM2* variant c.1493G>A [p.Arg498Gln]. WES results for Patient 2 are provided in Data Sheet 3 (WES ID: WES-190033286), who carries the *GPSM2* variant c.122G>A [p.Arg41His] (Figures 1A,B). Although the two variants are classified as “variants of uncertain significance” (VUS) according to the guidelines of the American Society of Human Genetics, the SIFT and PolyPhen-2 databases predicted that both *GPSM2* point mutations are more likely to be damaging (Supplementary Table S5). The human *GPSM2* gene is located on chromosome 1 and contains 15 exons, 14 of which encode proteins. The full-length transcript is 7,152 bases long and encodes 684 amino acids. Its full-length protein contains 6 TPR protein domains spanning amino acids 62–355 from the N-terminus to the C-terminus and 4 GoLoco protein domains at amino acids 489–650. WES revealed that the mutation site carried by Patient 1, *GPSM2*c.1493G>A, [p.Arg498Gln], is located within any known protein domain, whereas the mutation site carried by Patient 2, *GPSM2*c.122G>A, [p.Arg41His], is located in the GoLoco protein domain (Figure 1C). According to the conservation analysis of the *GPSM2* protein and comparison of the amino acid mutation sites identified in the patients, the amino acids at both mutation sites are highly conserved across major species (Supplementary Figure S2). Analysis using the STRING database revealed that human *GPSM2* interacts most strongly with INSC, whereas mouse *GPSM2* protein interacts most strongly with both NUMA1 and INSC. This difference may reflect the fact that the interaction between GPSM2 and NUMA1 has thus far been reported only in mice (Supplementary Figure S3).

### 3.2 Knockdown of *GPSM2* may induce the generation of aneuploid germ cells

**FIGURE 2:**
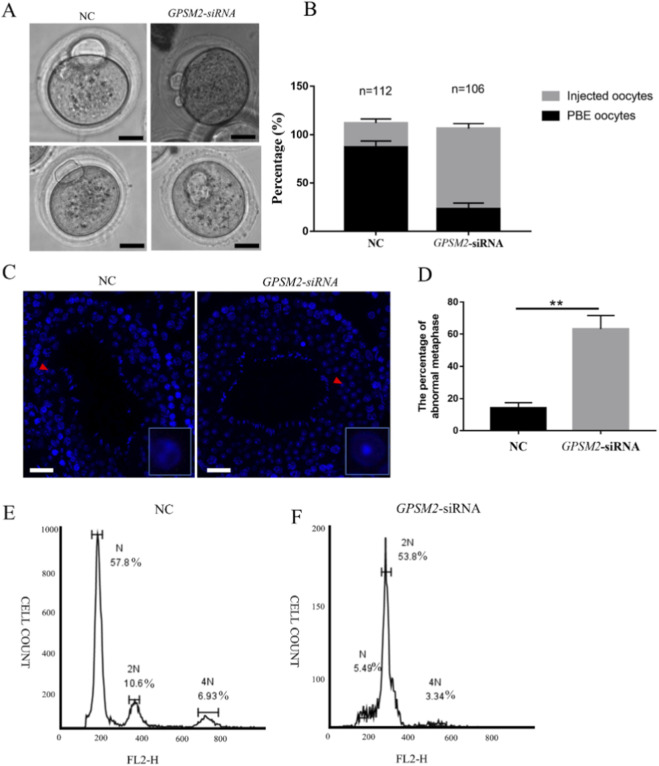
Knockdown of *GPSM2* may induce the generation of aneuploid germ cells. **(A)** A PB1 extruded from GV oocytes 13 h after siRNA injection. PB1, first polar body. Bar = 50 μm. **(B)** Statistical analysis of the total number of injected GV-stage mouse oocytes and first polar body extrusion. Injected oocytes, microinjected GV-stage mouse oocytes; PBE oocytes, oocytes exhibiting polar body extrusion. **(C)** DAPI staining of testicular sections from mice in the *GPSM2*–siRNA and NC groups. The “red triangle” indicates secondary spermatocytes. The lower right corner shows the secondary spermatocyte indicated by the “red arrow”. Bar = 50 μm **(D)**. Quantification of chromosome nondisjunction events during metaphase in secondary spermatocytes. n = 5, *p* < 0.05. **(E,F)** Flow cytometric analysis of the *GPSM2*–siRNA and NC groups (n = 5). Haploid (N), diploid (2N), and tetraploid (4N) cell populations are shown.

Interfering with *GPSM2* in mouse germinal vesicle (GV) oocytes can disrupt MI spindle organization (Guo and Gao, 2009). The interference efficiency of *GPSM2* siRNA was greater than 70% in GC-2 cells and mouse seminiferous tubules (Supplementary Figure S4). To investigate the effect of *GPSM2* knockdown on chromosome separation during germ cell meiosis, we first injected siRNA into mouse GV-stage oocytes and observed their phenotype after they were cultured to the MII stage. Only approximately 23% of the oocytes in the *GPSM2*–siRNA group could excrete the first polar body, and approximately 80% of the GV mouse oocytes in the NC group could successfully excrete the first polar body, suggesting that knocking down the expression of *GPSM2* in mouse oocytes may lead to the production of secondary oocytes with abnormal chromosome numbers; that is, aneuploid or polyploid secondary oocytes are produced (Figures 2A,B). To explore the causes of the abnormal chromosome number in spermatocytes of mouse testes after *GPSM2* interference, we examined testis sections from the two groups of mice following DAPI staining. Compared with the negative control (NC) group, approximately 60% of the secondary spermatocytes in the *GPSM2*–siRNA group were arrested at metaphase and failed to complete the meiotic division from secondary spermatocytes to spermatids. In contrast, only approximately 20% of the secondary spermatocytes in the NC group exhibited this phenomenon; these cells may have been in the metaphase stage of the second meiotic division, with chromosomes aligned on the equatorial plate. Approximately 80% of the secondary spermatocytes in the NC group displayed chromosomes migrating toward opposite poles of the cell (Figures 2C,D). Flow cytometry revealed that the percentages of haploid (N), diploid (2N), and tetraploid (4N) cells were 5.49%, 53.8%, and 3.34%, respectively, in the *GPSM2*–siRNA group and 57.8%, 10.6%, and 6.93%, respectively, in the NC group. Compared with those in the NC group, the number of haploid sperm cells in the *GPSM2*–siRNA group was significantly lower, and the number of diploid cells was significantly greater. These findings suggest that knockdown of *GPSM2* may lead to chromosome nondisjunction during metaphase in secondary spermatocytes of the mouse testis, resulting in a significant increase in the number of diploid cells, which are likely secondary spermatocytes arrested during chromosome segregation (Figures 2E,F). These results suggest that knocking down *GPSM2* may interfere with chromosome separation during sperm and egg maturation, increasing the probability of germ cell development with abnormal chromosome numbers.

### 3.3 Interference of *GPSM2* in the seminiferous tubules leads to oligoasthenozoospermia in mice

**FIGURE 3:**
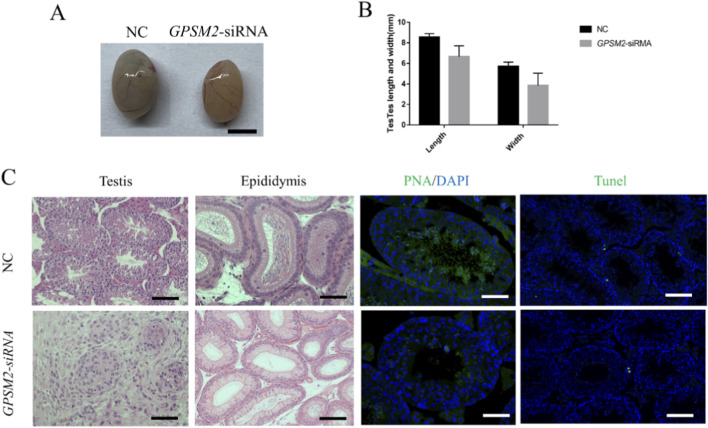
Phenotypes of the testis and epididymis in the *GPSM2*–siRNA and NC groups (n = 5; bar = 50 μm). **(A)** Mouse testes. **(B)** The length or width of the mouse testicles was statistically analyzed using the t-test method. (n = 5; unit: millimeters). **(C)** HE staining of paraffin-embedded sections of mouse testis and epididymis and PNA and TUNEL assays of mouse testis.

In this study, the length and width of the testis in the *GPSM2*–siRNA group were reduced compared with those in the NC group (Figures 3A,B). HE staining of the testes of the two groups of mice revealed that a large number of spermatocytes, spermatids, round spermatids, and elongating/elongated spermatids were missing in the *GPSM2*–siRNA group, and cell populations arranged from the wall of the seminiferous tubules to the center of the tubules according to spermatogony–spermatocyte–spermatid–spermatozoa were lacking. The phenotype of the NC group was consistent with that of the reported WT mouse testes. HE staining of the epididymis revealed a severe lack of mature, long-shaped sperm and round spermatids in the *GPSM2*–siRNA group, and the whole epididymis was very empty. The phenotype of the NC group was consistent with that of the WT mice, which was filled with a large number of mature sperm with well-developed heads and long, slender tails. PNA staining of the testes of the two groups revealed that the acrosome with sperm cells in the *GPSM2*–siRNA group was very rare, while the acrosome signal (green fluorescence signal) in the NC group was abundant, indicating that the acrosome-developed sperm cells were normal and abundant. TUNEL assays of testicular sections from the two groups of mice revealed no significant apoptotic events in the seminiferous tubules (Figure 3C). These results indicated that knockdown of *GPSM2* significantly reduced the number of spermatocytes in the mouse testis and ultimately reduced the number of sperm in the epididymis. This decrease was not caused by spermatocyte apoptosis.

### 3.4 Interference with the *GPSM2* gene did not affect spermatogenic events before chromosome segregation during MI in spermatocytes

**FIGURE 4:**
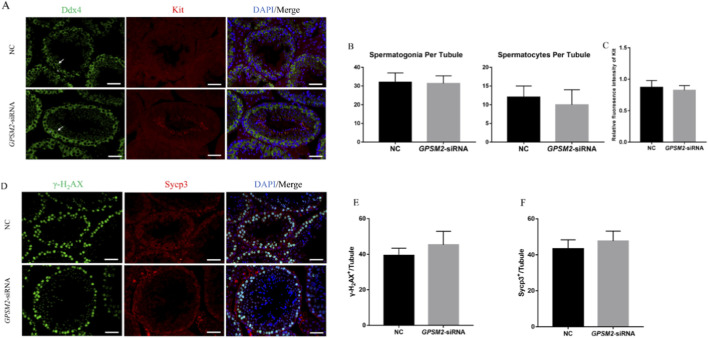
Immunofluorescence images of DXD44, Kit, γH2AX, and Sycp3 in testis sections from the GPSM2–siRNA and NC groups (n = 3; bar = 50 μm). **(A)** Immunofluorescence images of mouse testis sections; primary spermatocytes are indicated by “white arrows,” and spermatogonia are labeled with “white asterisks.” **(B)** Spermatogonia per tubule; spermatocytes per tubule. **(C)** Statistical plot of the relative fluorescence expression of the Kit marker. **(D)** Immunofluorescence images of mouse testis sections; **(E)** γH2AX+/tubule (statistical plot of cells with positive fluorescence for the γH2AX marker per seminiferous tubule). **(F)** Statistical plot of Sycp3+/tubule (cells with positive fluorescence for the Sycp3 marker per seminiferous tubule).

In this study, several marker proteins were selected from the published literature for immunofluorescence detection in testicular paraffin-embedded sections to explore the important biological events of *GPSM2* in the regulation of spermatogenesis. First, DDX4, a marker protein specific to spermatogenic cells, is commonly used to distinguish spermatogenic cells from somatic cells (Raz, 2000). Immunofluorescence staining revealed that the majority of the spermatogenic cells in the testes of *GPSM2*–siRNA and NC groups were intact, especially the primary spermatocytes, as indicated by the “white arrow,” which had a large number and complete cell structure, and the spermatogonia marked by the “white asterisk” had a normal number and complete cell structure (Figures 4A,B). Interference with *GPSM2* did not significantly affect the number of spermatogenic cells. Kit is a spermatogonial marker (Nakagawa et al., 2010), and little difference in the red fluorescence intensity of Kit protein labeling was detected in the seminiferous tubules of the *GPSM2*–siRNA and NC group mice (Figures 4A,C). These findings indicated that interference with *GPSM2* expression did not significantly affect the number of cells in the spermatogonial pool. Immunofluorescence assays using Plzf (Buaas et al., 2004; Costoya et al., 2004) and Stra8 (Anderson et al., 2008) protein markers confirmed that knockdown of *GPSM2* did not affect spermatogonial self-renewal or spermatogonial differentiation during spermatogenesis (Supplementary Figures S5A–C). Immunofluorescence assays using GFRα1 (Anderson et al., 2008) and Sox9 (Sun et al., 2022) protein markers in mouse testis sections confirmed that knockdown of *GPSM2* did not affect the self-differentiation of SSCs or the number of Sertoli cells (Supplementary Figures S5D–F).

To test whether the interference of *GPSM2* affects homologous chromosome association events and chromosomal DNA double-strand breaks in the interphase of spermatocytes, two protein markers, Sycp3 and γH2AX, were selected for immunofluorescence staining of mouse testis paraffin sections. Sycp3 is a marker of the chromosome association complex in primary spermatocytes during interphase (Zhang et al., 2021). As determined by the red fluorescence signal of Sycp3 in the testis sections of the *GPSM2*–siRNA and NC groups, the similar intensity of fluorescence signals in the two groups and a sufficient number of labeled primary spermatocytes indicated that the interference of *GPSM2* did not affect homologous chromosome association events in primary spermatocytes during interphase. γH2AX is a histone protein and a marker of chromosome breakage events (Zhang et al., 2021). In this study, the green fluorescence signal of γH2AX was similar between the two groups, the number of labeled spermatocytes was sufficient, and the cellular structure remained intact, indicating that *GPSM2* interference did not affect chromosome breakage events during meiotic interphase in spermatocytes (Figures 4D–F).

### 3.5 Knocking down *GPSM2* may interfere with the localization and block chromosome segregation

**FIGURE 5:**
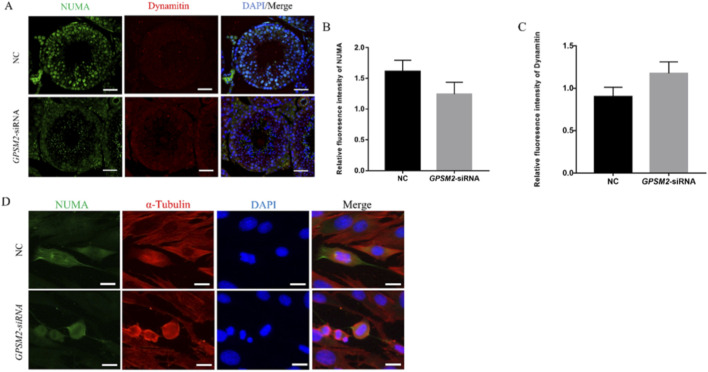
Immunofluorescence images of NUMA and dynamitin proteins in mouse testicular sections and statistical graphs of fluorescence intensities. **(A)** Immunofluorescence images of mouse testis sections. (n = 5; bar = 50 μm). **(B)** Statistical graph of the relative fluorescence expression levels of the NUMA protein marker. **(C)** Statistical graph of the relative fluorescence expression levels of the dynamitin protein marker (n = 3; bar = 50 μm). **(D)** Immunofluorescence images of NUMA- and α-tubulin-stained GC-2 cells (n = 3; bar = 50 μm).

The N-terminal TPR domain of the GPSM2 protein binds to the NUMA–dynactin–dynein complex during mitosis, whereas the C-terminus of the GPSM2 protein binds to the Gαi protein anchored to the inner membrane of the cell. Metaphase of cell division, when chromosomes are uniformly arranged in the equatorial plate: Gαi pulls on GPSM2 to coordinate the NUMA–dynactin–dynein complex, which pulls spindle microtubules toward the cell poles. This is used to guide chromosome segregation to obtain a normal number (Kiyomitsu and Cheeseman, 2012). Therefore, in the present study, we investigated whether GPSM2 proteins similarly bind NUMA proteins to participate in the regulation of normal chromosome segregation during meiosis. In the NC group, the GPSM2 protein was widely distributed in the spermatogonia, spermatocytes, and spermatids, whereas the α-tubulin protein fluorescence signal did not significantly change in the testicular sections of the two groups and remained normal. These findings indicated that the interference of *GPSM2* did not cause serious damage to testicular tissue structure (Figure 5A). Immunofluorescence staining of NUMA protein and dynamitin, a subunit of dynactin–dynein protein complex, in testicular sections from the two groups revealed that, compared with that in the NC group, in the *GPSM2*–siRNA group, the green fluorescence signal of NUMA was weakened in all cell layers of spermatogonia, spermatocytes, and spermatids, indicating that GPSM2 interference may affect the normal expression of NUMA protein. The red fluorescence intensity of the dynamitin protein did not significantly differ between the two groups, indicating that GPSM2 interference did not significantly affect the localization or expression of the dynactin–dynein complex protein (Figures 5A–C).

**FIGURE 6:**
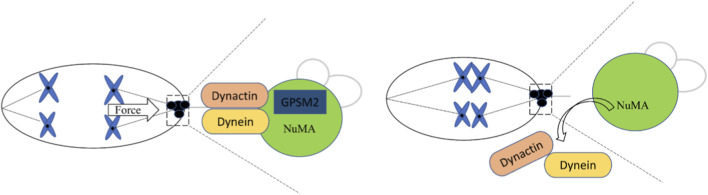
*GPSM2* participates in the regulation of meiotic chromosome segregation in spermatocytes.

Given that the subcellular localization of spermatocyte target proteins could not be clearly observed in mouse testicular sections, GC-2 cells were chosen as the cellular model for this study: GC-2 cells remain in a “mitotic” mode for more than 30 generations of cell division, and no genomic information was integrated and altered since only two non-mouse plasmids were transferred. The proteins involved in the completion of the so-called “mitosis” are all authentic regulatory proteins during spermatocyte meiosis. Therefore, in this study, GC-2 cells (approximately 105 cells) were treated with *GPSM2*–siRNA or *NC*–siRNA for 48 h. Immunofluorescence assays were performed on cell slides, and NUMA and α-tubulin (spindle marker proteins) were used as markers to observe the localization of regulatory proteins during chromosome segregation. In this study, we found that GC-2 cells in the *GPSM2*–siRNA group were arrested at metaphase, with chromosomes neatly aligned at the center of the equatorial plate and unable to move toward the poles. No colocalization signals between NUMA and α-tubulin were observed at the spindle poles. However, the GC-2 cells in the NC group had moved to the cell poles under the traction of the complex protein, and the cell membrane continued to divide. A clear colocalization signal between NUMA and α-tubulin was observed at the poles of the spindle (Figure 5D). Previous studies have reported that the GPSM2 protein can interact with the NUMA protein *in vitro* (Zhu et al., 2011). These results suggest that GPSM2 may also bind to the NUMA protein and localize to the spindle poles, guiding chromosomes aligned at the equatorial plate toward the poles and ultimately facilitating chromosome segregation with the correct chromosome number (Figure 6).

## 4 Discussion

Fetal aneuploidy is among the main causes of RSA (Larsen et al., 2013). In this study, WES of patients with RSA with an abnormal number of abortive products revealed that two female patients carried *GPSM2* missense point mutations. Patients with Chudley–McCullough syndrome often carry homozygous *GPSM2* mutations (Yariz et al., 2012; Doherty et al., 2012; Walsh et al., 2010), but the relationships among *GPSM2* mutations, aneuploid embryos, and gametophyte formation have not been reported. Therefore, the purpose of this study was to explore the role of *GPSM2* in regulating chromosome separation during sperm‒egg maturation. *GPSM2* is involved not only in cell mitosis but also in the regulation of oocyte meiosis (Zhu et al., 2011; Du et al., 2001; Guo and Gao, 2009). Therefore, using mouse spermatocytes and oocytes as models, we demonstrated that knockdown of *GPSM2* can lead to abnormal chromosome segregation in mouse spermatocytes and significantly increase the number of diploid spermatogenic cells. At the poles of the spindle during the metaphase of mitosis, the N-terminal TPR domain of the GPSM2 protein binds specifically to the C-terminus of the NUMA protein, leading the NUMA protein to bind to the dynactin–dynein complex proteins to localize the stellate microtubule-associated chromosomes to the poles (Kiyomitsu and Cheeseman, 2012). In this study, immunofluorescence staining revealed that chromosomes were arrested and failed to segregate during metaphase in GC-2 cells when *GPSM2* was knocked down and that the NUMA protein could not localize to the spindle poles. Moreover, *in vivo* IP and *in vitro* pull-down assays revealed that the N-terminus of *GPSM2* could bind to the C-terminus of the NUMA protein (Zhu et al., 2011). Therefore, the NUMA protein may form a complex with the GPSM2 protein to bind to the dynactin–dynein complex. Under the traction of the GPSM2 protein to the poles of the cell, the NUMA–dynactin–dynein complex protein guides the spindle microtubules to pull chromosomes to the poles, ensuring that the chromosomes reach the correct position and complete the chromosome separation process of meiosis (Figure 6). The pathogenic mechanism may involve the inability of the NUMA protein to interact with sufficient GPSM2 protein, resulting in its improper localization to the spindle poles.

Owing to the risk of off-target effects associated with gene-editing technology, this study did not construct an animal model that exactly replicated the missense mutation of the *GPSM2* gene identified in the patients. Although siRNA-mediated interference disrupting *GPSM2* function cannot fully recapitulate the effects of a point mutation, it was used to simulate the pathogenic consequences of *GPSM2* loss of function on the generation of chromosomal aneuploidy in reproductive cells. Based on predictions of the harmfulness of the *GPSM2* missense mutation carried by the patients using the SIFT, PolyPhen-2 and PROVEAN databases, combined with the literature reporting that *GPSM2* is involved in regulating the process of mitotic chromosome segregation, we conducted a series of experimental studies on the chromosome segregation regulation process of *GPSM2* during sperm and egg formation to confirm that the GPSM2 protein also plays an important role in regulating chromosome segregation during meiosis. Our research findings have provided new insights into the potential pathogenesis of gene function impairment caused by the *GPSM2* missense mutation, which may increase the risk of recurrent miscarriage with aneuploidy. We have also identified potential pathogenic loci that deserve special attention. These research results also offer certain research direction guidance for reducing the clinical risk of recurrent miscarriage.

## Data Availability

The original contributions presented in the study are included in the article/Supplementary Material, further inquiries can be directed to the corresponding author. The WES data has been uploaded to the National Gene Bank of China (https://db.cngb.org/) under project number CNP0008044.
